# Gambling behaviors in Israeli adults: findings from a nationally representative sample

**DOI:** 10.1186/s13584-025-00733-0

**Published:** 2025-12-15

**Authors:** Belle Gavriel-Fried, Inbar Malka

**Affiliations:** The Bob Shapell, School of Social Work, Tel Aviv University, Tel Aviv, 69978 Israel

**Keywords:** Gambling behavior, Risk factors, Israel, Representative sample

## Abstract

Globally, 46.2% of all adults report having gambled in the previous 12 months. While most experience no negative repercussions from gambling, individuals who are highly engaged in gambling are at greater risk of problem gambling (PG). Studies point to the psycho-social, environmental, and socio-demographic risk factors associated with gambling and PG, and the associated harm to individuals, families, and society, making it a public health concern worldwide. Israel has a relatively regulated conservative gambling market where casinos and electronic gambling machines are banned, and online gambling is only permitted for sports betting. Nevertheless, Israel has a relatively high percentage of PG. This study was designed to characterize involvement in gambling behavior during the previous year in non-gamblers, low-frequency, and high-frequency gamblers as a function of socio-demographic, health, and psycho-social risk and protective factors, and differentiate between problem and non-problem high-frequency gamblers. A representative sample of 3224 Israeli Jews and Arabs filled in an online questionnaire in 2022. The findings showed that 50.7% were non-gamblers, 33.8% were low-frequency gamblers, and 15.5% were high-frequency gamblers. Compared to non-gamblers, low-frequency and high-frequency gamblers were associated with Jewish ethnicity, low levels of financial self-efficacy and neighborhood cohesion, a greater likelihood to smoke and drink, and having a significant other (family/social network member) with perceived excessive gambling. Male gender and traditional self-perceived religiosity were associated with both low-frequency and high-frequency gamblers as well, but to a greater extent with high-frequency than low-frequency gamblers. Online gambling, stress, low financial self-efficacy, using social welfare allowances for gambling, having a significant other who engages in perceived excessive gambling, and identifying as an Israeli Arab were all associated with PG in high-frequency gamblers. These findings suggest that despite its conservative regulated market, gambling in Israel exceeds international averages. The similarities between low-frequency and high-frequency gamblers in terms of risk factors may hint at a type of gambling normalization. Regulatory reforms informed by public health policies are recommended to decrease access to gambling, including banning online gambling and curbing special gambling offers timed to coincide with welfare payments. Awareness campaigns, culturally sensitive prevention programs are recommended, as well as studies to monitor gambling harm.

## Introduction

Gambling is a major public health concern worldwide [1, 2]. Gambling is widespread globally, with large proportions of people engaged in various types of gambling [3]. Although recreational gambling tends not to lead to substantial problems or have detrimental effects, a subset of individuals, typically those who are highly involved in gambling, have an increased risk of adverse consequences [3–5]. Gambling can lead to financial difficulties [6], physical and mental health concerns [7] including attempted suicide [8], as well as strained social relationships [9]. The detrimental social and economic repercussions of gambling extend beyond the individual gambler to impact their significant others and society as a whole [3].

Official gambling policies take a public health approach that prioritizes the public good and social justice [10]. This approach seeks to reduce risk and promote well-being by targeting specific problems across diverse populations [11] which calls for an integrative and whole-system approach that balances individual responsibility (e.g., treatment, self-exclusion) and structural factors (e.g., regulating gambling availability) to prevent harm [12]. This type of multidimensional framework aims to identify bio-psychosocial, financial, and cultural risk factors [13, 14] and address harmful behavior along a continuum to support prevention, recovery, and effective policy implementation [15].

Previous studies have reported associations between gambling and a number of socio-demographic and psychosocial factors. Males, younger age groups, and individuals with fewer years of education and low socio-economic status (SES) tend to be more involved in gambling than women, older age groups, and individuals with more years of education and higher SES [4, 16–19]. Higher levels of religiosity are also associated with less frequent gambling [20]. Other socio-demographic variables associated with gambling frequency include race, income and marital status, with studies showing that ethnic minorities and the least affluent gamble more often [18, 21]. Research indicates that more frequent gamblers tend to be divorced, widowed, or cohabiting males [22].

Nevertheless, the findings for income and marital status are mixed with respect to individuals defined as problem gamblers, in that both high and low income have emerged as risk factors, alongside a range of marital statuses [23]. Gambling tends to spike on welfare benefit payout days, suggesting that gambling frequency is related to cash from this source [24].

Higher frequencies of gambling are also correlated with a greater likelihood of alcohol consumption, binge drinking, tobacco use and more numerous episodes of poorer mental health [25, 26]. Anxiety and depression symptoms have also been related to an increase in gambling frequency [27]. Other factors that positively associate with gambling include proximity to lottery venues, as well as friends and family members who gamble or view it positively [19, 28]. Monetary gain has often been shown to be a driver for gambling [29] and predicts gambling problems over time [30]. Studies report significant associations between gambling and gambling problems or harms [2, 5, 31, 32]. Studies have found that mixed-mode gamblers, i.e., individuals who engage in both online and land-based gambling, gamble the most, and that online-only and mixed gamblers have more gambling problems than land-based only gamblers [33]. These findings reinforce the claim that the specific features of online gambling make it a significant risk factor [34].

Israel constitutes an intriguing setting for examining gambling patterns and behaviors, given the features of its gambling market [35]. Current Israeli legislation authorizes specific types of gambling under the auspices of two designated authorized operators: The Israeli Lottery “Mifal Hapais” that operates various types of daily and weekly lotteries, and the Israel Sports Betting Board (ISBB) (“Winner-Toto”) that runs on-site and online sports betting. All other forms of gambling, including private gambling activities, are illegal [36]. Thus, the gambling arena in Israel is relatively conservative in terms of types of gambling [37], but still offers a variety of regulated gambling options to the public, albeit without land-based gambling such as the casinos or slot machine arcades commonly seen in other countries [38]. Although a significant proportion of all gambling in Israel is legal [35, 36], previous studies report that 8.6%−13% of all gamblers engage in illegal gambling activities [35, 39].

In 2016, the Israeli government adopted the recommendations of two committees that examined the gambling arena in Israel and published two reports. The first was an inter-ministerial committee appointed by the government, which was headed by a representative of the Ministry of Justice and included representatives from several government ministries. It published a report presenting a series of recommendations for regulating legal gambling in the country [40] that included reducing overexposure to gambling, preventing addiction, reducing risk to vulnerable groups, combating the appeal of gambling advertising and marketing, as well as ways to deal with the risks related to the potential use of gambling for criminal activity (money laundering), and the potential for criminal elements to infiltrate gambling operations. During the final stages of the hearings of the first committee, the Israel Finance Ministry set up a parallel committee to optimize the gambling market and the profit allocation model [41], probably in an attempt to regain control over the gambling market to further protect the State’s economic/budgetary interests. This committee recommended limiting the overhead expenses of the gambling bodies, restricting the scope of the legal gambling market, and changing the mechanisms for allocating the profits. The recommendations of the two committees were approved by a governmental decision that same year [42]. Several recommendations made by the first committee have been carried out (outlawing of horse race betting, allocation of funds for treatment and for independent research on gambling). However, concerns on the part of the Ministry of Finance related to the considerable loss of State revenue from gambling [43], associated with the tendency in Israeli policies to resist regulation in general in Israel, and the ongoing political instability in Israel have stalled the implementation of the other key recommendations.

This aligns with the observations by critics of gambling policies worldwide, who argue that these policies have failed to translate the acknowledgment of gambling as a public health concern into comprehensive and effective policies [10]. This failure not only reinforces the power and influence of the gambling industry but also undermines public acknowledgment of gambling as a legitimate public health issue [44]. In the Israeli context, more pressing security and other political concerns, as well as the factors outlined above, have tended to overshadow the risks of gambling in Israel.

The gambling sector’s revenue from lotteries and betting in 2023 amounted to approximately $2.38 billion for “Mifal Hapais” [45] and $930.2 million for “Winner-Toto” [46]. A recent representative epidemiological study on problem gambling (PG) in Israeli adults found that 49.3% had gambled in the previous year. Of these individuals, 32.6% were classified as non-problem gamblers, 10.4% as low-risk gamblers, and 6.3% as moderate-risk and problem gamblers [35]. A recent, non-representative sample reported a slightly higher percentage [47].

Given the relatively high percentage of individuals with gambling-related problems in Israel, coupled with the association between high gambling involvement and PG [2], it is thus crucial to pinpoint the risk and protective factors associated with the entire spectrum of gambling consumption as manifested by frequency of gambling, in particular, since individuals who do not meet the clinical threshold for Gambling Disorder (GD) can still experience significant harm [48].

Based on a nationally representative sample of Israeli citizens, the current study was designed to explore gambling in Israel, and specifically to (1) assess the frequencies of involvement in different forms of gambling and the participants’ motivation for engaging in them, (2) characterize involvement in gambling behaviors into non-gamblers, low-frequency (LF) and high-frequency (HF) gamblers as a function of socio-demographic, health and psycho-social risk and protective factors [3], identify the sociodemographic, health, and psychosocial factors associated with levels of gambling involvement and 4) identify the differences between HF problem and non-problem gamblers. It was hypothesized that HF gamblers and those with gambling problems would score lower on measures of protective characteristics and resources and higher on measures of risk/negative factors than LF gamblers or respondents who reported no gambling activity. The findings can thus serve to frame recommendations for policymakers in Israel.

## Method

### Sample and procedure

This study is part of a broader research project examining gambling behavior in Israeli adults in a web-based representative random sample drawn from the Israeli population. This panel was extracted by the B.I and the Lucille Cohen Institute for Public Opinion Research at Tel Aviv University in compliance with methodological guidelines for web-based research as outlined by the National Opinion Research Center at the University of Chicago and the PEW Research Institute for studies pertaining to Israeli society [49]. The panel was structured to encompass one segment for Israeli Jews and one segment for Israeli Arab citizens of Israel, mirroring the ethnonational stratification in Israel. Israeli Jews were selected at random from the population registry database, which made it possible to retrieve their phone numbers. Israeli Arabs were randomly recruited from household databases. The distribution of gender and age in the Israeli Jewish panel members closely mirrored that of the Jewish population in Israel, with the exception of individuals aged 70 and over, who were underrepresented. The panel had an overrepresentation of college graduates and an underrepresentation of individuals defining themselves as Ultra-Orthodox. Comparable administrative data on the gender and age distribution in Israeli Arabs were not readily available [49].

An invitation e-mail, SMS or both, and six reminders containing the survey link (in Hebrew for Israeli Jews and in Arabic for Israeli Arabs) were sent to all panel members between July and September 2022. The panel consisted of 10,499 Israeli Jews and 1,302 Israeli Arabs. The response rates were 30.9% for Israeli Jews and 11.7% for Israeli Arabs, for a total of 3,080 Israeli Jews and 164 Israeli Arabs. All the panel members signed an informed consent. This study was approved by the Tel Aviv University ethics committee (#0002764-3).

## Measures

### Frequency of gambling behavior

 A composite measure was created specifically for this study covering the 11 most common types of gambling in Israel (e.g., Lotto, scratch cards) along with an additional item labeled “other” to include unspecified forms of gambling. Participants were requested to specify how often they engaged in each type of gambling in the previous year on a five-point Likert scale (1 = ‘Not at all’ 5 = ‘once a week or more’).

The respondents were grouped into three categories based on their scores: those who responded negatively to all items (i.e., a total score of 1) were classified as non-gamblers, those at or below the 70th percentile were classified as LF gamblers, and those above the 70th percentile were classified as HF gamblers. The total mean for the scores for the 12 gambling types was 1.12 (on a scale of 1 to 5) with a SD of 0.19. Based on the SD, respondents scoring −1SD from the mean corresponded to the minimum score of 1 on the scale and were classified as non-gamblers. Respondents scoring up to +1SD from the mean, corresponding to the 70% percentile (among those who gamble) were classified as LF gamblers, and respondents above the 70% percentile were classified as HF gamblers. 

In addition, the participants were asked to indicate the medium through which they typically engaged in gambling when presented with six distinct options (e.g., internet on personal computer, internet/online on mobile phone, gambling venues in Israel). Respondents were classified as online gamblers if they indicated one or more forms of online play.

### Problem gambling severity index (PGSI)

 The PGSI is a nine-item scale that ranks PG severity [50]. The scale ranges from 0 (‘never’) to 3 (‘almost always’). Individuals are divided into four groups: 0 = ‘Non-problem gamblers’; 1–2 = ‘Low-risk gamblers’; 3–7 = ‘Moderate-risk gamblers’; and 8+ = ‘Problem gamblers’. In the current study, ‘moderate-risk gamblers’ and ‘problem gamblers’ were combined, as done in previous epidemiological studies [51, 52]. The Cronbach’s alpha was 0.80.

### Gambling motivation

A single question, developed by Williams et al. [53] asking the participants to select their primary reason for gambling from eight predefined choices (e.g., to win money, to socialize), and an “Other” category.This variable was used for descriptive purposes alone.

### Short gambling harm screen (SGHS)

The SGHS is a 10 item yes/no scale that evaluates the extent of harm caused by gambling in a range of life domains experienced by the individual in the previous year, such as harm to financial and emotional well-being [54]. The total score is calculated by summing the ‘yes’ responses, with higher scores indicating greater severity of gambling-related harm.

### Perceived excessive gambling in a significant other

A one-item [55] yes/no question asking the participants to indicate whether someone in their life (family/social network) in their opinion, gambled too much.

### Material deprivation

This six-item yes/no scale evaluates the extent to which the respondents experienced material deprivation [56] with respect to goods and services. These six items pertain to the capacity or inability to procure medicine or receive medical care, have enough to eat, the wherewithal to make home repairs, buy new clothes, pay utility bills, and school supplies and expenditures. Higher scores indicate higher material deprivation.

### Socioeconomic cluster

The socioeconomic cluster of the respondents’ residential locality was determined from the Israel Central Bureau of Statistics’ 2019 locality classification [57] that ranges from 1 (low cluster) to 10 (high cluster).

### Welfare payments

The participants were asked whether they had used their social welfare allowance (e.g., disability, child benefits) for gambling purposes in the previous 12 months, on a scale from 1 (‘never’) to 5 (‘always’). Due to the small number of participants who responded positively, the score was dichotomized into – 0 = ‘never’, 1 = ‘rarely to always’.

### Patient Health Questionnaire-4 (PHQ-4)

This ultra-brief scale evaluates levels of depression and anxiety: two items assess generalized anxiety (GAD-2) and two assess depression (PHQ-2) [58]. Respondents rate their responses on a four-point scale ranging from ‘no symptoms’ (= 0) to ‘almost daily symptoms’ (= 3), where a total score of 3 or more is indicative of risk of depression or anxiety. In the current study, the Cronbach alpha for the GAD-2 was 0.87 and the PHQ-2 was 0.56.

### Subjective stress

Respondents were asked to rank a single item: “In the previous month, to what extent, if at all, have you felt stressed?“. They responded to this item on a scale ranging from 0 to 10, with 10 denoting the highest stress.

### Tobacco consumption

A single item was utilized: “Over the previous month, how many cigarettes or tobacco products did you smoke a day?” Responses ranged from 0 – ‘I didn’t smoke’ to 6 – ‘30 or more’. The score was dichotomized into yes/no.

### Alcohol consumption

A single item was utilized: “Over the previous month, how many times did you drink at least one or more alcoholic beverages (e.g., beer, wine, etc.) other than for religious purposes?” Responses ranged from 0 – ‘Never’ to 6 – ‘30 times or more’. The score was dichotomized to yes/no.

### Financial self-efficacy

This 6-item scale evaluates individuals’ perceived level of self-efficacy in financial matters [59] and taps into respondents’ ability to address financial challenges. For example: ‘I find it challenging to adhere to my budget when faced with unexpected expenses’. Responses range from 1 (complete disagreement) to 4 (strong agreement). After reversing all items, the responses were aggregated. The overall score ranges from 4 to 24, with a higher total indicating higher financial self-efficacy. The Cronbach’s alpha was 0.80.

### Neighborhood cohesion

This five-item scale assesses the respondents’ perception of social cohesion in their neighborhood [60]. Participants evaluate cohesion on a five-point Likert scale ranging from 1(‘strongly disagree’) to 5 (‘strongly agree’). A higher total indicates greater neighborhood cohesion. The Cronbach’s alpha was 0.70.

### Demographic variables

The respondents indicated their gender, age, level of education, marital status, ethnonational affiliation (Israeli Jewish, Israeli Arab), and self-perceived religiosity (secular, traditional, religious, and Ultra-Orthodox). In the results, ethnonational affiliation is designated as ‘ethnicity’.

#### Data analysis

The data were analyzed with SPSS (ver. 29) and were weighted by ethnonational affiliation (Israeli Jewish/Arab) and level of education. Descriptive statistics were calculated for the variables, the total sample, and by gambling frequency. Frequencies are presented as unweighted values. Percentages, means and standard deviations are presented as weighted values. The bivariate analyses included multinomial logistic regression models, with gambling frequency as the dependent variable (no gambling, LF gamblers, HF gamblers, and socio-demographic and psycho-social risk and protective factors, as well as gambling-related factors as the independent variables). Types of gambling were described by gambling frequency. Then, based on the descriptive comparisons, two multinomial logistic regression models were evaluated: [1] LF and HF gambling vs. no gambling with demographic and other non-gambling related variables as the independent variables [2], LF and HF gambling, including gambling-related variables as the independent variables, in addition to all others. Due to the multitude of independent variables, both regression models were calculated in a stepwise manner, such that only the significant variables were entered into the final model. Categorical variables were defined on the basis of the descriptive results, as dummy variables. Finally, all the study variables for the HF gamblers were compared, using logistic regression models, while differentiating between non-problem gamblers and moderate-risk/problem gamblers. Significant independent variables were entered into a multiple stepwise logistic regression model to assess their relative contribution to moderate-risk vs. PG.

## Results

### Descriptive statistics and comparisons

As shown in Table 1, roughly half of the respondents indicated that they did not gamble at all (*n* = 1618, 50.7%), one-third were categorized as LF gamblers (*n* = 1141, 33.8%), and the remainder were categorized as HF gamblers (*n* = 485, 15.5%). Most gamblers played the Lotto (about 73%), followed by scratch cards, Chance, Pais 777, 123 - instant win ticket gambling, legal sports betting, and on the stock market (about 14% to 41%). Illegal gambling had the lowest rates of stated involvement (10% or less).

**Table 1:** Frequencies of gambling involvement as a function of gambling types, in descending order (*N* = 1626) in the past 12 months

Gambling types	Total (*n* = 1626) *n*(%)	LF gamblers (*n* = 1141) *n*(%)	HF gamblers (*n* = 485) *n* (%)
Lotto (once or twice a week raffle)	1156 (72.6)	735 (66.6)	421 (85.7)
Scratch cards	625 (41.1)	336 (30.3)	289 (64.5)
Chance, Pais 777, 123 – (Instant win ticket Gambling)	245 (21.4)	61 (10.6)	184 (45.0)
Legal (regulated) sport betting	195 (14.8)	53 (5.7)	142 (34.7)
Stock market	284 (14.2)	154 (10.9)	130 (21.3)
Card games	164 (9.8)	48 (4.1)	116 (22.0)
Slot machines	105 (8.2)	40 (3.5)	65 (18.5)
Table games	106 (7.7)	38 (4.5)	68 (14.5)
Illegal sports betting	39 (5.0)	9 (3.3)	30 (8.7)
Bingo	31 (1.8)	6 (0.4)	25 (4.8)
Horse racing	8 (0.5)	3 (0.2)	5 (1.0)
Other	87 (4.8)	64 (5.1)	23 (4.2)

LF- Low-frequency; HF – High-frequency

The respondents reported that their main reason for gambling was financial (“win money”, *n* = 958, 53.4% of *n* = 1794), followed by “excitement and entertainment or fun” (*n* = 332, 18.5%). The other reasons were less common: “to compete or for the challenge” (*n* = 66, 3.7%), “support worthy causes promoted by the gambling industry” (*n* = 67, 3.7%), “to escape, relax, or relieve stress” (*n* = 26, 1.4%), “to socialize” (*n* = 34, 1.9%), “to develop my skills” (*n* = 34, 1.9%), “it makes me feel good about myself” (*n* = 16, 1.0%). Of the respondents, 14.4% (*n* = 259) reported “other”.

### Differences between gambling frequencies

Tables 2, 3 and 4 present comparisons between levels of gambling frequency (non-gamblers, LF and HF gamblers) as a function of socio-demographic and psycho-social risk and protective factors, as well as gambling-related factors as the associated variables. Relative to non-gamblers, LF gamblers were more likely to be male, with no high school diploma, more likely to be Jewish, and less likely to be religious. They were more likely to be in a higher socioeconomic cluster and less likely to report material deprivation. They were less likely to be classified in the clinical range for depression and anxiety and scored lower on stress. They were more likely to smoke and drink. They scored lower on financial self-efficacy, lower on neighborhood cohesion, and were more likely to have a member of their family/social network whom they perceived as involved in excessive gambling.

Relative to non-gamblers, the HF gamblers were more likely to be males, less likely to be young adults and more likely to be in their thirties or older adults (65+), with no high school diploma or academic degree, more likely to be Jewish, more likely to be traditional and less likely to be religious. They were more likely to smoke and drink. They scored lower on financial self-efficacy, lower on neighborhood cohesion, and were more likely to have an excessive gambler in their family or social network.

Finally, HF gamblers, relative to LF gamblers, were more likely to be males, in their thirties or older (65+), and less likely to be in their thirties or middle-aged [40–64], more likely not to have a high school diploma or an academic degree, less likely to be secular and more likely to be traditional. They were more likely to be in a lower socioeconomic cluster and report material deprivation. They were more likely to be classified into the problem levels of gambling, and to engage in online gambling. They scored higher on gambling-related harm, used social welfare allowances for gambling, and ranked lower on financial self-efficacy. They were more likely to be classified in the clinical range for depression and anxiety and score higher on stress.

**Table 2:** Socio-demographic and socioeconomic characteristics by gambling frequency *N* = 3244)

Variable	Total (*N* = 3244) *n*(%) M(SD)	Non- gamblers (*n* = 1618) *n*(%) M(SD)	LF gamblers (*n* = 1141) *n*(%) M(SD)	HF gamblers (*n* = 485) *n*(%) M(SD)	χ^2^ (*p*)	LF vs. Non- gamblers OR (95%CI)	HF vs. Non- gamblers OR (95%CI)	HF vs. LF gamblers OR (95%CI)
Gender (male)	1409 (46.4)	573 (38.0)	529 (52.1)	307 (61.9)	111.18 (*p* <.001)	**1.78** **(1.52**,** 2.07)**	**2.66** **(2.17**,** 3.27)**	**1.50** **(1.21**,** 1.86)**
Age group								
18–29	740 (22.9)	394 (24.1)	249 (23.1)	97 (18.3)	27.13 (*p* <.001)	0.95 (0.79, 1.13)	**0.71** **(0.55**,** 0.91)**	**0.75** **(0.57**,** 0.98)**
30–39	737 (23.1)	359 (22.7)	246 (21.1)	132 (28.6)		0.91 (0.75, 1.09)	**1.36** **(1.09**,** 1.70)**	**1.50** **(1.18**,** 1.91)**
40–64	1396 (43.7)	675 (43.4)	526 (46.3)	195 (38.8)		1.12 (0.96, 1.31)	0.82 (0.67, 1.01)	**0.73** **(0.59**,** 0.91)**
65+	371 (10.3)	190 (9.7)	120 (9.5)	61 (14.3)		0.98 (0.76, 1.28)	**1.56** **(1.16**,** 2.10)**	**1.58** **(1.15**,** 2.18)**
Level of education								
No high-school diploma	490 (29.1)	191 (25.6)	186 (30.2)	113 (38.1)	33.81 (*p* <.001)	**1.25** **(1.06**,** 1.49)**	**1.79** **(1.45**,** 2.21)**	**1.42** **(1.14**,** 1.78)**
High school diploma, no academic degree	1111 (36.3)	550 (37.3)	390 (35.2)	171 (35.1)		0.91 (0.78, 1.07)	0.91 (0.74, 1.12)	0.99 (0.80, 1.24)
Academic degree	1643 (34.6)	877 (37.1)	565 (34.6)	201 (26.8)		0.90 (0.77, 1.05)	**0.62 (0.50**,** 0.77)**	**0.69 (0.55**,** 0.87)**
Marital status (married, in a relationship)	2240 (67.4)	1090 (65.7)	796 (69.1)	354 (69.4)	4.70 (*p* =.095)	1.17 (0.99, 1.38)	1.19 (0.96, 1.47)	1.01 (0.81, 1.28)
Ethnicity (Jewish)	3066 (79.1)	1506 (74.2)	1092 (84.4)	468 (83.5)	48.78 (*p* <.001)	**1.87** **(1.54**,** 2.28)**	**1.77** **(1.36**,** 2.30)**	0.94 (0.71, 1.26)
Self-perceived religiosity								
Secular	1565 (40.4)	815 (40.1)	544 (43.0)	206 (35.7)	39.69 (*p* <.001)	1.13 (0.97, 1.32)	0.83 (0.67, 1.02)	**0.73 (0.59**,** 0.91)**
Traditional	1031 (39.4)	438 (36.4)	394 (39.1)	199 (49.4)		1.12 (0.96, 1.31)	**1.71** **(1.40**,** 2.09)**	**1.52** **(1.23**,** 1.88)**
Religious, very religious	648 (20.2)	365 (23.4)	203 (17.9)	80 (14.9)		**0.71** **(0.44**,** 0.75)**	**0.57** **(0.44**,** 0.75)**	0.80 (0.60, 1.08)
Material deprivation (yes)	617 (25.5)	328 (28.3)	197 (19.3)	92 (29.8)	34.27 (*p* <.001)	**0.61** **(0.51**,** 0.73)**	1.07 (0.86, 1.33)	**1.76 (1.38**,** 2.24)**
Socioeconomic cluster, M(SD)	5.69 (2.31)	5.52 (2.37)	5.96 (2.29)	5.66 (2.12)	23.42 (*p* <.001)	**1.09** **(1.05**,** 1.12)**	1.02 (0.98, 1.07)	**0.94** **(0.90**,** 0.99)**

LF- Low-frequency; HF – High-frequency Note. 95%CI that do not include the value of 1 are significant

**Table 3:** Gambling-related variables by gambling frequency (*N* = 1626)

Variable	Total (*N* = 1626) *n*(%) M(SD)	LF gamblers (*n* = 1141) *n*(%) M(SD)	HF gamblers (*n* = 485) *n*(%) M(SD)	χ^2^ (*p*)	HF vs. LF gamblers OR (95%CI)
PGSI					
Non-problem gamblers	1169 (66.1)	909 (75.6)	260 (45.6)	136.56 (*p* <.001)	**0.27 (0.22**,** 0.34)**
Low-risk gamblers	293 (21.0)	161 (15.8)	132 (32.3)		**2.57 (2.00**,** 3.29)**
Moderate-risk and problem gamblers	164 (12.9)	71 (8.7)	93 (22.0)		**2.97 (2.21**,** 4.01)**
Gambling-related Harm (0–10), M(SD)	0.86 (1.69)	0.66 (1.43)	1.31 (2.09)	48.20 (*p* <.001)	**1.24 (1.16**,** 1.32)**
Use allowances (yes)	29 (2.7)	13 (1.1)	16 (6.2)	29.90 (*p* <.001)	**5.80 (2.95**,** 11.38)**
Online gambling	299 (18.6)	124 (10.8)	175 (35.7)	132.86 (*p* <.001)	**4.60 (3.54**,** 5.99)**

Bolded numbers indicate significant associations. LF- Low-frequency; HF - High-frequency Note. 95%CI that do not include the value of 1 are significant

**Table 4:** Psycho-social and health risk and protective factors by gambling frequency (*N* = 3244)

Variable	Total (*N* = 3244) *n*(%) M(SD)	Non- gamblers (*n* = 1618) *n*(%) M(SD)	LF gamblers (*n* = 1141) *n*(%) M(SD)	HF gamblers (*n* = 485) *n*(%) M(SD)	χ^2^ (*p*)	LF vs. non- gamblers OR (95%CI)	HF vs. non- gamblers OR (95%CI)	HF vs. LF gamblers OR (95%CI)
Depression, clinical (≥ 3)	900 (32.0)	481 (34.2)	295 (27.2)	124 (35.1)	17.57 (*p* <.001)	**0.72** **(0.61**,** 0.85)**	1.04 (0.84, 1.28)	**1.44** **(1.15**,** 1.81)**
Anxiety, clinical (≥ 3)	622 (23.2)	337 (25.0)	197 (19.6)	88 (25.4)	12.63 (*p* <.001)	**0.73** **(0.61**,** 0.88)**	1.02 (0.81, 1.28)	**1.40** **(1.09**,** 1.79)**
Stress, M(SD)	4.48 (2.81)	4.60 (2.70)	4.17 (2.84)	4.77 (3.06)	22.01 (*p* <.001)	**0.95** **(0.92**,** 0.97)**	1.02 (0.99, 1.06)	**1.08** **(1.04**,** 1.12)**
Tobacco per day (yes)	791 (27.4)	322 (22.3)	308 (31.4)	161 (35.3)	46.39 (*p* <.001)	**1.60** **(1.35**,** 1.90)**	**1.90** **(1.53**,** 2.36)**	1.18 (0.95, 1.48)
Alcohol per month (yes)	1871 (50.0)	872 (42.0)	684 (56.2)	315 (62.9)	93.17 (*p* <.001)	**1.78** **(1.52**,** 2.07)**	**2.34** **(1.90**,** 2.87)**	**1.32** **(1.06**,** 1.63)**
**Protective**								
Financial self-efficacy, M(SD)	16.10 (3.69)	16.48 (3.59)	15.89 (3.72)	15.30 (3.78)	44.14 (*p* <.001)	**0.96** **(0.94**,** 0.98)**	**0.92** **(0.89**,** 0.94)**	**0.96** **(0.93**,** 0.99)**
**Environmental**								
Neighborhood cohesion, M(SD)	17.05 (3.52)	17.37 (3.51)	16.86 (3.46)	16.41 (3.58)	33.53 (*p* <.001)	**0.96** **(0.94**,** 0.98)**	**0.93** **(0.90**,** 0.95)**	0.96 (0.94, 0.99)
Significant other who gambles excessively (yes)	312 (10.6)	118 (6.8)	110 (13.9)	84 (15.9)	52.21 (*p* <.001)	**2.21** **(1.71**,** 2.85)**	**2.56** **(1.88**,** 3.47)**	1.16 (0.86, 1.55)

Bolded numbers indicate significant associations. LF- Low-frequency; HF - High-frequency Note. 95%CI that do not include the value of 1 are significant

## Multinominal and logistic regression models

Based on the descriptive comparisons, several multinomial and logistic regression models were evaluated. The first examined HF and LH gamblers vs. non gamblers, with demographic and other non-gambling related variables as independent factors. The second examined HF vs. LF gamblers, including gambling-related variables as independent factors, in addition to all others (Table 5). The third logistic regression modeled HF problem gamblers vs. HF gamblers without gambling problems (Table 6). Given the multitude of independent variables, the regression models were calculated in a stepwise manner, such that only the significant independent factors were entered into the final model. Categorical variables were defined on the basis of the descriptive results as dummy variables: gender (1-male, 0-female), age group (1 = 30–39 and 65+, 0 = 18–29 and 40–64), level of education - first (1- no high school diploma, 0-high school diploma and higher), level of education − (1-academic, 0-lower than academic), level of religiosity (1-traditional, 0-secular and religious), ethnicity (1-Jewish, 0-non Jewish), material deprivation (1-yes, 0-no), clinical classification of depression (1-yes, 0-no), tobacco per day (1-yes, 0-no), alcohol per month (1-yes, 0-no), low and moderate-high risk gamblers (1-yes, 0-non problem gamblers), online gambling (1-yes, 0-no).

Both regression models in Table 5 were significant. For LF and HF gamblers vs. non gamblers, χ^2^ [26] = 510.12, *p* <.001, Nag. R^2^ = 0.17; and for LF vs. HF gambling: χ^2^ [9] = 279.76, *p* <.001, Nag. R^2^ = 0.23. The regression model in Table 6 was significant as well: χ^2^ [6] = 124.06, *p* <.001, Nag. R^2^ = 0.43.

The results showed that compared to non-gamblers and LF gamblers, HF gamblers were more likely to be males, in their thirties or older (65+), and have lower levels of education (compared to non-gamblers, HF gamblers were more likely not to have a high school diploma, and compared to LF gamblers, HF gamblers were more likely not to have a college degree). Compared to non-gamblers and LF gamblers, HF gamblers were more likely to be Jewish, and be traditional in terms of religiosity.

Compared to non-gamblers, HF gamblers had higher stress levels, were more likely to smoke cigarettes and drink, reported lower financial self-efficacy and neighborhood cohesion, and had someone who gambles excessively in their family/social network. Compared to LF gamblers, HF gamblers were more likely to report material deprivation, higher stress levels, meet the criteria for PG, and were more likely to gamble online. The comparison between LF gamblers and non-gamblers yielded similar results, with the exception that LF gamblers reported lower levels of stress than non-gamblers.

It should be noted that male gender (*p* =.003), and traditional self-perceived religiosity (*p* =.001) were more strongly associated for HF gamblers than for LF gamblers. No differences in the strength of the association were found for ethnicity (*p* =.084), low levels of financial self-efficacy (*p* =.999), low neighborhood cohesion (*p* =.597), greater likelihood to smoke (*p* =.650) and drink (*p* =.075), or a network member with perceived excessive gambling (*p* =.737).

**Table 5:** Multinomial regression models for gambling frequency, with socio-demographic and psycho-social risk and protective factors (*N* = 3244)

Variable	LF gamblers vs. non-gamblers OR (95%CI)	HF gamblers vs. non-gamblers OR (95%CI)	HF gamblers vs. LF gamblers OR (95%CI)
Gender (male)	**1.67 (1.41**,** 1.98)**	**2.57 (2.04**,** 3.22)**	**1.39 (1.09**,** 1.78)**
Age group (30–39 and 65+)	0.93 (0.78, 1.10)	**1.50 (1.21**,** 1.87)**	**1.68 (1.31**,** 2.14)**
Level of education- (no high school diploma)	1.19 (0.98, 1.45)	**1.49 (1.16**,** 1.91)**	**--**
Level of education- (academic)	--	**--**	**0.75 (0.57**,** 0.97)**
Ethnicity (Jewish)	**2.19 (1.72**,** 2.79)**	**3.14 (2.26**,** 4.37)**	**2.15 (1.51**,** 3.08)**
Level of religiosity (traditional)	**1.35 (1.13**,** 1.62)**	**2.15 (1.72**,** 2.70)**	**1.40 (1.09**,** 1.79)**
Material deprivation (yes)	**0.56 (0.45**,** 0.70)**	0.82 (0.62, 1.08)	**1.43 (1.05**,** 1.94)**
Clinical classification of depression (yes)	**0.77 (0.64**,** 0.94)**	0.90 (0.70, 1.15)	**--**
Level of stress	**0.96 (0.93**,** 1.00)**	**1.02 (0.98**,** 1.07)**	**1.06 (1.01**,** 1.10)**
Tobacco per day (yes)	**1.47 (1.21**,** 1.77)**	**1.37 (1.08**,** 1.74)**	**--**
Alcohol per month (yes)	**1.42 (1.19**,** 1.69)**	**1.85 (1.46**,** 2.34)**	**--**
Financial self-efficacy	**0.91 (0.89**,** 0.94)**	**0.91 (0.88**,** 0.94)**	**--**
Neighborhood cohesion	**0.95 (0.93**,** 0.98)**	**0.94 (0.91**,** 0.97)**	**--**
Significant other who gambles excessively	**1.98 (1.51**,** 2.59)**	**2.13 (1.53**,** 2.96)**	**--**
Low and moderate-high risk gamblers (yes)	**--**	**--**	**2.83 (2.21**,** 3.64)**
Online gambling (yes)	**--**	**--**	**3.70 (2.78**,** 4.92)**

LF- Low-frequency; HF - High-frequency Note. 95%CI that do not include the value of 1 are significant

Finally, to test for differences between HF non-problem gamblers and HF problem gamblers, all the variables were compared among HF gamblers (*n* = 485), between non-problem gamblers (*n* = 260), and moderate-risk/problem gamblers (*n* = 93), according to their PGSI scores (Table 2). Most of the differences were significant in the expected direction: age group (*p* =.004), level of education (*p* =.037), marital status (*p* <.001), religion (*p* <.001), religiosity (*p* =.005), material deprivation (*p* <.001), socio economic cluster (*p* =.001), use of social welfare allowances (*p* <.001), online gambling (*p* <.001), depression (*p* <.001), anxiety (*p* <.001), stress (*p* <.001), tobacco use (*p* =.003), financial self-efficacy (*p* <.001), neighborhood cohesion (*p* <.001), reports of excessive gamblers in their family/social networks (*p* =.011) (Tables 2, 3 and 4).

To assess the contribution of these variables to the risk for moderate/PG, a logistic regression was conducted. Note that gambling harm was not included in this regression model because it overlapped with moderate/PG, thus possibly masking the potential contribution of other variables. Due to the small sample size and the multitude of independent variables, the regression was calculated as a stepwise model rather than hierarchically. As shown in Table 6, HF gamblers who were Israeli Arabs, used social welfare allowances for gambling, gambled online, reported higher levels of stress, lower financial self-efficacy, and whose family/social networks included someone who engaged in excessive gambling were at a higher risk of moderate/PG.

**Table 6:** Stepwise logistic regression model for the risk of moderate/problem gambling, as a function of socio-demographic and psycho-social risk and protective factors (*N* = 353)

Variable	Moderate/problem gamblers vs. non-problem gamblers		
	OR	p	95%CI
Ethnicity (Jewish)	0.27	0.035	0.08, 0.91
Used social welfare allowance (yes)	16.06	0.002	2.70, 95.39
Online gambling (yes)	4.25	< 0.001	2.40, 7.54
Stress	1.12	0.029	1.01, 1.25
Financial self-efficacy	0.89	0.009	0.82, 0.97
Significant other who gambles excessively (yes)	2.60	0.009	1.27, 5.32

## Discussion

These findings provide key insights into the national Israeli context and more broadly on the risk and protective factors associated with gambling. The findings showed that 49.3% of all Israelis in the panel reported having gambled in the previous year, compared to 46.2% globally [4]. This finding is surprising given the regulated conservative gambling market in Israel that has no casinos or EGMs, and where online gambling is restricted to the sports betting authority. This suggests that a conservative market does not necessarily result in a decrease in gambling. This argument is further supported by the findings that the respondents who gambled reported significantly more engagement in legal than illegal gambling. This is suggestive of the draw of the flourishing Israeli gambling industry which, despite its structure, provides appealing, diverse daily lottery and sports betting opportunities through extensive advertising that produces high revenues for these outlets [45, 46]. Sports betting in Israel is legal in land-based venues and also online. Effective and more restrictive regulatory policies in Israel should thus be enacted to monitor the legal gambling industry in terms of its funding, availability, and accessibility.

The primary reported reasons for gambling were monetary, followed by thrill and excitement. These rationales are consistent with findings from national surveys in the U.K [61]., Australia [62], and a recent study conducted in Israel [63]. Financial motives have been linked to gambling frequency [29] and predict gambling problems over time [30]. These findings prompt a number of recommendations concerning the potential prevention and treatment programs in Israel.

Jewish ethnicity, low levels of financial self-efficacy and neighborhood cohesion, greater likelihood to smoke and drink, and a network member with perceived excessive gambling were associated with both LF and HF gamblers. Although male gender and traditional (in terms of self-perceived religiosity) had stronger associations for HF gamblers than for LF gamblers, they both were significantly associated with both LF and HF gamblers. Bearing in mind the cross-sectional design of this study, which precludes drawing causal conclusions, these findings underscore the multifaceted nature of factors related to gambling [28], and suggest that individuals who gamble (regardless of frequency) may be prone to similar social, economic, and psychological influences that are associated with gambling. This may reflect the normalization of gambling in everyday life [44]. In particular, the similarities in risk factors across low and high-frequency gamblers may indicate that gambling is perceived as a socially acceptable and widespread behavior, rather than one confined to specific “at-risk” groups. This argument is supported by the findings that being male, Jewish, and traditional, all of which are attributes that reflect dominant groups in Israeli society [64], are associated with individuals who gamble (regardless of frequency) but not with individuals who experience gambling problems. Future longitudinal studies should monitor the transition from low to high involvement and PG and explore the mechanisms driving these risk factors over time.

PG and online gambling characterized the respondents who were classified as HF gamblers. These traits are consistent with findings on PG [5] and online gambling [19, 33], and highlight the potential risks inherent to online gambling associated with PG. Thus, frequent and less frequent gamblers are likely to have similar risk or protective factors, but where they play – in particular online gambling – may have a crucial role.

The findings differentiating HF non-PG from HF with PG help point to the key risk factors for PG in Israel. These characteristics included ethnicity; namely, being an Israeli Arab, using social welfare allowances for gambling, online gambling, high levels of stress, low financial self-efficacy, and having an excessive gambler in one’s family or social network. Previous findings have also reported a link between ethnic minorities and PG [21, 65]. In the case of Israeli Arabs who face structural inequality, social exclusion, and limited access to social and economic resources [66], PG seems to have emerged here as yet another social problem. In this context, gambling may serve as a maladaptive mechanism to cope with stress, alienation, or perceived lack of control over one’s life. The use of social welfare allowances for gambling provides compelling evidence for the relationship between poverty and PG. It is consistent with studies indicating that lower socio-economic status is significantly associated with more frequent gambling [21] and that various poverty-related indicators, such as low income and housing instability, are linked to PG [67], including for individuals receiving social welfare benefits [68]. It also supports findings showing that gambling revenues in Israel are significantly higher on welfare stipend payout days [69]. This finding is concerning. While the State provides financial support to those in need, some of this aid apparently ultimately returns to State coffers through regulated gambling revenues. This paradox exposes the multiple and contradictory roles of governments that function simultaneously as regulators and as beneficiaries of gambling. It prompts concerns regarding the State’s ethical conduct [70], and underscores the need to establish an independent regulatory body.

Financial self-efficacy clearly differentiated between HF PG and HF non-PG gamblers (in addition to its association with LF and HF). Financial self-efficacy is the belief in one’s ability to successfully confront financial hurdles, such as meeting financial objectives or sticking to a budget [59, 71], and involves a cognitive construct rather than simply financial incentives. The findings here thus contribute to the substantial body of literature that conceptualizes self-efficacy as a shield against engagement in risky behaviors [72], including gambling [73], where financial self-efficacy was shown to exemplify a specific form of self-efficacy.

Stress characterized HF gamblers, and was also associated with PG. This lends weight to consistent findings indicating that gambling is both a stressor and a way to escape stress, and that altered stress physiology may predispose towards GD [74]. This risk factor deserves attention in Israel, where the public is exposed to continuous stressors.

Having a family or a network member engaged in perceived excessive gambling was associated with all individuals who gamble, regardless of frequency and severity. This highlights the potential influence of the social environment on gambling behavior. It supports findings in studies implementing Social Learning Theory [75] which show that individuals learn to gamble through social networks and social interactions [76], that higher-risk groups and individuals classified as problem gamblers are surrounded by other gamblers [19], and that gambling behavior and its associated harms are frequently normalized through social connections [77]. Note that given the cross-sectional design of previous studies as well as in this study, the directionality of causality cannot be determined; i.e., whether social influence drives gambling behavior or whether individuals who gamble or have gambling problems connect to people with the same interests.

Finally, the findings identified the 30 to 40 age group and the 65 and over group as more likely to be HF but not problem gamblers. This is consistent with findings showing that frequent gambling peaks in the 30 s [78], and a recent Australian survey showing that regular gamblers tend to be 50 and older and retirees [79]. More studies should explore the gambling patterns of these age groups and monitor them over time.

### Implications for practice and policy

The evolving discourse in the gambling field has shifted from an emphasis on individual responsibility for gambling to a broader public health perspective that addresses societal impacts and risk factors, and advocates for comprehensive intervention strategies [10, 44, 80]. The results showing a relatively high frequency of individuals who are HF gamblers in this national representative study, in addition to the association between HF gamblers and PG, and the association between PG and variables such as ethnonational affiliation, the use of social welfare allowance, and stress underscore the importance of implementing various strategies as advocated by the public health approach, which specifically consists of integrating gambling issues into a national action plan, and utilizing a multidisciplinary approach to address specific individual problems in conjunction with broader regulatory measures [81].

Below, a number of recommendations are put forward, consistent with calls in other countries to acknowledge gambling as a societal issue that engages collective responsibility rather than being viewed solely as an individual concern: (a) One workable possibility would be for the state of Israel to establishing an independent and adequately empowered regulatory body to promote an integrated public health policy; (b) Policy makers could weigh the value of raising public awareness of gambling-related issues through educational initiatives and public campaigns, including limiting advertising and marketing to reduce public exposure [82, 83]. These campaigns have the potential to de-normalizing gambling behavior [48]; (c) Thoughts could be given to developing culturally-sensitive prevention and treatment programs for Israeli Arabs that would include linguistically appropriate psycho-educational programs dealing with cultural values and beliefs about gambling which may tend to encourage gambling as a traditional activity [84]; (d) Steps could be taken at the government level to evaluate the possibility of mandating account-based gambling systems that incorporate universal daily, monthly, and annual loss limits. This would create a safer regulated environment by enabling monitoring of gambling patterns and reducing reliance on voluntary self-control measures [85]; (e) The government could evaluate the feasibility and impact of prohibiting online betting on legal websites, given the proliferation of online sports gambling [86] and its inherent risks [33, 34]; (f) The government could evaluate the feasibility of limiting lotteries held on benefit payout days or restricting the amount of money people on benefits can spend on gambling to make it difficult or prevent welfare recipients from gambling on these days. This would be similar to a policy enacted in Austria, where income assessment or a history of bankruptcy limit gambling access [87]; (g) Similarly, the government could consider issuing fewer permits for lottery kiosks in economically disadvantaged areas where more people are on welfare; (h) Population-level longitudinal studies to monitor gambling behavior and associated harm over time.

Finally, the data for this study were collected before the October 7th, 2023 war but were analyzed and written in its aftermath, during a period in which Israeli society is continuing to confront its most significant national trauma in the past five decades. Research conducted globally points to associations between trauma and the emergence of post-traumatic stress disorder and more problematic gambling [88]. Conflicts may lead to an escalation in the number of individuals resorting to gambling as a strategy to handle depression, stress, and anxiety. The gambling industry in Israel should consider shouldering greater social responsibility and taking steps to reduce the prevalence of individuals in Israel who are more susceptible to the adverse consequences of gambling than in the past.

### Limitations

The cross-sectional design of this study precludes drawing causal conclusions such that the findings should only be interpreted in terms of differences and associations. Although widely used in public health research, this study is also based on self-reports, which can be subject to bias given imperfect recall or deliberation [89]. Future studies should use a mixed-methods design to overcome this limitation. Gambling motivations were assessed across all gambling types, even though it is well-established that motivations may differ according to the specific form of gambling. Works could consider examining these measures separately by gambling type to capture more granular differences. The response rate for the Israeli Arab panel was relatively low (11.7%), leading to an underrepresentation of Israeli Arabs compared to their proportion in the general population. This aligns with findings from population surveys in other countries that include ethnonational minorities [90]. Studies should use different and more diverse recruitment methods to target this population. Despite these limitations, this first epidemiological study on gambling behavior used a representative sample of the Israeli population, a strength that can pave the way for future cross-sectional and/or longitudinal studies monitoring gambling patterns in Israeli society.

## Data Availability

Data available on request from the first author.
